# Extracorporeal cardiopulmonary resuscitation dissemination and integration with organ preservation in the USA: ethical and logistical considerations

**DOI:** 10.1186/s13054-023-04432-7

**Published:** 2023-04-18

**Authors:** Tamar Schiff, Christian Koziatek, Erin Pomerantz, Nichole Bosson, Robert Montgomery, Brendan Parent, Stephen P. Wall

**Affiliations:** 1 Department of Population Health, NYU Langone Health, 227 E 30th St, New York, NY 10016 USA; 2 Ronald O. Perelman Department of Emergency Medicine, NYU Langone Health, New York, NY USA; 3 NYU Langone Transplant Institute, NYU Langone Health, New York, NY USA; 4 Lewis Katz School of Medicine at Temple University, Philadelphia, PA USA; 5 Los Angeles County EMS Agency, Santa Fe Springs, CA USA; 6 Harbor-UCLA Medical Center and the Lundquist Research Institute, Torrance, CA USA; 7 David Geffen School of Medicine at UCLA, Los Angeles, CA USA; 8 Department of Surgery, NYU Langone Health, New York, NY USA

**Keywords:** Extracorporeal membrane oxygenation, Cardiopulmonary resuscitation, Organ donation, Organ preservation, Bioethics

## Abstract

Use of extracorporeal membrane oxygenation (ECMO) in cardiopulmonary resuscitation, termed eCPR, offers the prospect of improving survival with good neurological function after cardiac arrest. After death, ECMO can also be used for enhanced preservation of abdominal and thoracic organs, designated normothermic regional perfusion (NRP), before organ recovery for transplantation. To optimize resuscitation and transplantation outcomes, healthcare networks in Portugal and Italy have developed cardiac arrest protocols that integrate use of eCPR with NRP. Similar dissemination of eCPR and its integration with NRP in the USA raise novel ethical issues due to a non-nationalized health system and an opt-in framework for organ donation, as well as other legal and cultural factors. Nonetheless, eCPR investigations are ongoing, and both eCPR and NRP are selectively employed in clinical practice. This paper delineates the most pressing relevant ethical considerations and proposes recommendations for implementation of protocols that aim to promote public trust and reduce conflicts of interest. Transparent policies should rely on protocols that separate lifesaving from organ preservation considerations; robust, centralized eCPR data to inform equitable and evidence-based allocations; uniform practices concerning clinical decision-making and resource utilization; and partnership with community stakeholders, allowing patients to make decisions about emergency care that align with their values. Proactively addressing these ethical and logistical challenges could enable eCPR dissemination and integration with NRP protocols in the USA, with the potential to maximize lives saved through both improved resuscitation with good neurological outcomes and increased organ donation opportunities when resuscitation is unsuccessful or not in accordance with individuals’ wishes.

## Background

Extracorporeal membrane oxygenation (ECMO) was originally developed for intraoperative cardiopulmonary bypass. Since its introduction, this technology has been employed in continuously broader clinical applications including emergency and critical care [1]. Growing data supports that, in appropriately selected patients, using ECMO for cardiopulmonary resuscitation (eCPR) offers the prospect of improved survival with good neurological function compared to conventional resuscitation [2]. ECMO can also be used to selectively perfuse abdominal and thoracic tissues after circulatory death, termed normothermic regional perfusion (NRP), to improve transplantation outcomes [3, 4].

Since eCPR and NRP both employ ECMO technology, healthcare systems in Portugal and Italy have developed integrated eCPR/NRP protocols aiming to maximize lives saved by improving outcomes after cardiac arrests while also optimizing organs recovered postmortem for transplantation when resuscitation is unsuccessful or not in accordance with individuals’ wishes [5–7]. Investigations of eCPR in the USA have been reported and are ongoing [8–11], and both eCPR and NRP are selectively employed in clinical practice [12–15]. But broader dissemination of eCPR programs and integration of eCPR with NRP raise novel ethical and logistical issues in the USA, in large part because of the lack of a nationalized health system and an opt-in framework for organ donation. Wholly realizing the promise of such programs in the USA therefore requires transparent protocols that proactively address ethical considerations by directing equitable provision of evidence-based care, promoting public trust, and reducing conflicts of interest.

### ECMO for resuscitation

**Fig. 1:**
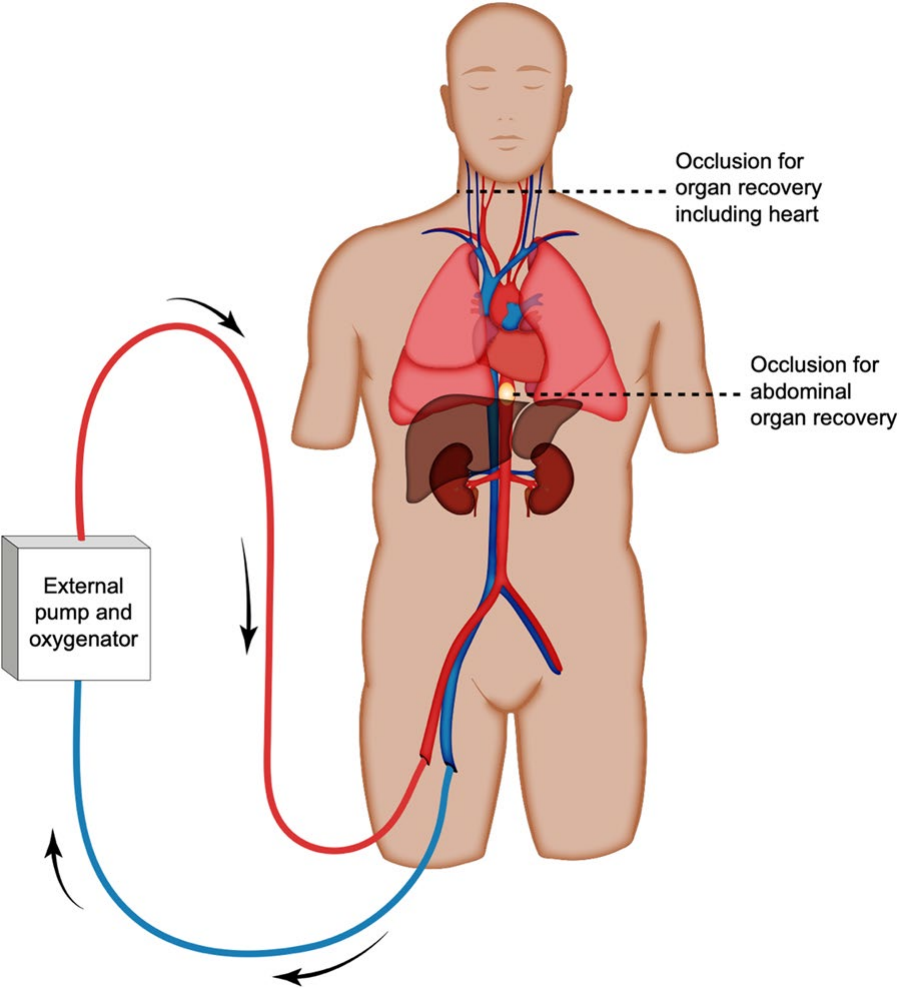
Use of ECMO circuit in eCPR, NRP for cDCD, and NRP for uDCD. *ECMO* extracorporeal membrane oxygenation; *eCPR* extracorporeal cardiopulmonary resuscitation; *NRP* normothermic regional perfusion; *cDCD* controlled donation after circulatory death; *uDCD* uncontrolled donation after circulatory death. The ECMO circuit consists of a venous access cannula, draining venous blood to a pump device, which pumps deoxygenated venous blood through an oxygenator membrane that also scavenges carbon dioxide and manages temperature. The oxygenated blood returns to a central artery via an arterial return cannula. In eCPR, the goal is to achieve return of spontaneous circulation with good neurological function. Critical to achieving that goal is providing circulation of oxygenated blood to the brain. In NRP, balloon occlusion in the aorta at the level of the diaphragm (to recover abdominal organs) or surgical ligation of blood vessels to the brain (to recover the heart) is performed to ensure natural progression of complete and irreversible loss of brain function postmortem

Over 500,000 adults in the USA suffer cardiac arrest annually [16]. Despite progress in conventional CPR, only 10.4% of patients who arrest outside of hospitals and 25.6% who arrest inside hospitals survive to discharge [16]. To improve prognosis for select candidates, a veno-arterial ECMO configuration can bypass the heart and lungs to circulate oxygenated blood throughout the body while underlying causes of cardiac arrest are identified and treated (Fig. 1) [2]. Importantly, unlike standard CPR, which can yield prolonged states of decreased blood flow resulting in ischemic damage, eCPR provides sufficient sustained perfusion to vital organs, including the brain [2]. Consequently, eCPR offers potential for improved survival with good neurological function, often defined in clinical trials as a cerebral performance category (CPC) score of 1 or 2 [17].

Studies employing eCPR report promising results [8, 18–21], but there remains a need for more robust data. While there are further trials underway, published investigations to date have been mostly single-centered and/or observational with inconsistent eligibility criteria [22–24]. As a recent example, a randomized trial of out-of-hospital cardiac arrest in Prague demonstrated 9.5% improvement in survival with good neurologic outcome at 180 days in patients who underwent intra-arrest transport, eCPR, and immediate invasive assessment and treatment, compared to those who received standard advanced cardiac life support. However, this result did not reach statistical significance, possibly due to the trial being underpowered [18]. While eCPR implementation in the USA has been relatively sparse to date, in part from constrained resources, need for specialized training, and high cost [10, 25], the prospect of markedly improving post-cardiac arrest outcomes warrants ardent investigation through clinical trials. Evolving data indicate that appropriate selection of patients who may benefit from eCPR and its rapid initiation correlate with improved outcomes [19, 25].

### ECMO for organ preservation (NRP)

There are over 100,000 individuals on organ transplant waitlists in the USA and approximately 12,000 of these patients become too sick for transplantation or die due to the organ shortage annually [26]. Presently, organ recovery in the USA occurs most often via donation after neurological death (DND) [26], despite neurological deaths accounting for only 2% of hospital deaths among adults [27]. Healthcare systems in the USA are therefore expanding organ recovery from donation after circulatory death (DCD). In controlled DCD (cDCD), eligible patients in whom cardiopulmonary support is determined futile or not aligned with their wishes have support withdrawn under monitoring [28]. After death determination and a “hands-off” period, during which the heart is monitored for autoresuscitation, organ recovery occurs (Table 1). While cDCD expansion contributed to an 11% increase in deceased donations in 2021 [26], opportunities remain woefully inadequate to meet transplant demand. Therefore, as in some European countries, the USA should consider how to ethically incorporate donation following unexpected deaths from cardiopulmonary arrest, termed uncontrolled DCD (uDCD) [29]. Estimates project uDCD could increase donation opportunities in the USA by about 10,000 to 22,000 cases annually [30, 31].

**Table 1:** The Modified Maastricht Classification of DCD [22]

Category		Definition
I Uncontrolled	Found dead IA. Out-of-hospital IB. In-hospital	Sudden unexpected circulatory arrest without any attempt of resuscitation by a medical team
II Uncontrolled	Witnessed cardiac arrest IIA. Out-of-hospital IIB. In-hospital	Sudden unexpected irreversible circulatory arrest with unsuccessful resuscitation by a medical team
III Controlled	Withdrawal of life sustaining therapy	Planned withdrawal of life-sustaining therapy; expected circulatory arrest
IV Uncontrolled/controlled	Circulatory arrest after neurological determination of death	Sudden circulatory arrest after neurological determination of death diagnosis during donor management but prior to planned organ recovery

In both cDCD and uDCD, there are several techniques for organ preservation. NRP uses a modified ECMO circuit to circulate oxygenated blood to donor organs within the body after death has been declared. Compared to rapid recovery techniques, NRP allows donor assessment and organ procurement under considerably lesser time constraints, and it may reduce iatrogenic injury to the recovered tissues [32]. Studies also suggest that using NRP in DCD minimizes warm ischemic damage and reduces some post-engraftment complications in transplanted kidneys and livers in comparison to standard in situ cold preservation and rapid recovery [3, 4, 33]. To limit perfusion to only the part of the body with viable transplantable organs, excluding the brain, initiating NRP postmortem requires either balloon occlusion of the thoracic aorta at the diaphragm for abdominal organ preservation or surgical ligation of the brain’s arterial supply to include preservation of the heart (Fig. 1) [3]. While potentially improving transplant outcomes and cost efficiency, NRP protocols face barriers in the USA due to opt-in donation policies and ambiguities in the legal definition of death reviewed below [34–39].

### Integrated eCPR/NRP protocols

Protocols using eCPR offer the potential to improve cardiac arrest outcomes, and organ preservation protocols employing NRP carry the prospect of enhanced transplant outcomes. Integrating these two applications of ECMO therefore presents the distinct opportunity to maximize clinical efficacy and resource efficiency. But integrated eCPR/NRP protocols also raise crucial new ethical concerns. Given that investigations of eCPR are ongoing, ethical considerations must be proactively explored and addressed in anticipation of the possibility of wider dissemination of this clinical practice in the USA. Moreover, we posit that for the sake of equity, patient autonomy, and efficiency, eCPR trials should be integrated with those of NRP where possible so that the benefit of using ECMO for both purposes can be better understood and developed.

**Fig. 2:**
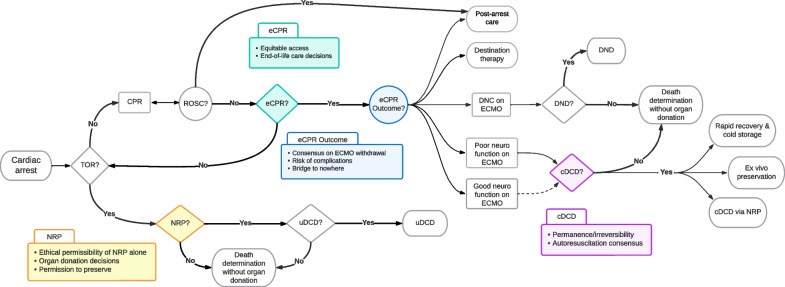
Concept map for integrating eCPR and uDCD programs within opt-in organ donation systems. *TOR* termination of resuscitation; *CPR* cardiopulmonary resuscitation; *ROSC* return of spontaneous circulation; *ECMO* extracorporeal membrane oxygenation; *eCPR* extracorporeal cardiopulmonary resuscitation via ECMO; *DNC* death by neurological criteria; *DND* donation after neurological death; *cDCD* controlled donation after circulatory death; *NRP* normothermic regional perfusion of organs via ECMO; *uDCD* uncontrolled donation after circulatory death. The figure presents a schematic overview of a clinical protocol for eCPR/NRP integration. Ovals designate starting/ending points, rectangles are processes, diamonds are decision points, circles represent uncertainty in outcomes. Critical steps are represented in colors which correspond to summary boxes highlighting prominent ethical and logistical concerns, which are discussed individually at length in the main text

Figure 2 presents a schematic overview of an integrated eCPR/NRP clinical protocol. Represented in the top arm of Fig. 2, eCPR is employed for clinically eligible patients in whom there is realistic hope of reversible and treatable causes of refractory cardiac arrest [6]. Following successful resuscitation, patients will go on to receive post-cardiac arrest care as clinically warranted. Should eCPR fail to achieve survival with good neurological function, DND [40] and cDCD opportunities should be offered to eligible candidates. When standard CPR is unsuccessful and eCPR is contraindicated, NRP should be offered to candidates eligible for uDCD, shown in the bottom arm of Fig. 2. The diagram in Fig. 2 is not intended to be a prescriptive algorithm for clinical implementation, but instead a map of the major steps and decision points in such algorithms to allow elucidation of critical ethical and logistical considerations that must be addressed to appropriately disseminate trials of eCPR and integrated eCPR/NRP protocols in the USA. The discussion that follows examines these key considerations with proposed recommendations to address them.

## Considerations in implementing eCPR protocols

### Equitable access

The geographic and demographic differences in resource availability among healthcare institutions in the USA have major impacts on access to and receipt of clinical interventions [41, 42]. Healthcare disparities are further amplified because the USA does not operate under a nationalized health system, unlike several countries in which eCPR protocols are being developed. Assuming eCPR research data continues to show benefit, eCPR protocols in the USA must dictate inter-hospital coordination, triaging, and transfer when essential therapies are selectively available, akin to regional systems for patients with myocardial infarctions or strokes, which ensure access to hospitals capable of performing emergent percutaneous vascular interventions [43, 44]. Given resource limitations, such as equipment and personnel required to operate ECMO circuits, treatment allocations should depend on availability, need, and potential benefit to recipients. Policies should include contingencies for triaging and/or reallocating ECMO circuits and personnel when resources are particularly scarce (e.g., respiratory viral pandemics) [45]. By iteratively assessing data on survival and outcomes, the clinical community should develop guidelines to hone optimal eligibility criteria and to encourage uniform practice and inter-institutional coordination, rather than varied decisions of individual hospital systems or clinicians.

### End-of-life care decisions

Currently, resuscitation discussions routinely involve questions concerning chest compressions, intubation, and use of certain medications. As efficacy and availability of novel resuscitation techniques and technologies improve, goals-of-care discussions likewise should be adapted. If eCPR is available, these discussions require explanations about cannulation and use of ECMO among possible interventions, the potential benefits and complications of eCPR, and its evidence-driven implementation. Discussions must also convey that eCPR allocation is determined solely by clinical indication and never influenced by organ donation status.

## Considerations in uncertainty of eCPR outcomes

### Consensus on ECMO withdrawal

**Fig. 3:**
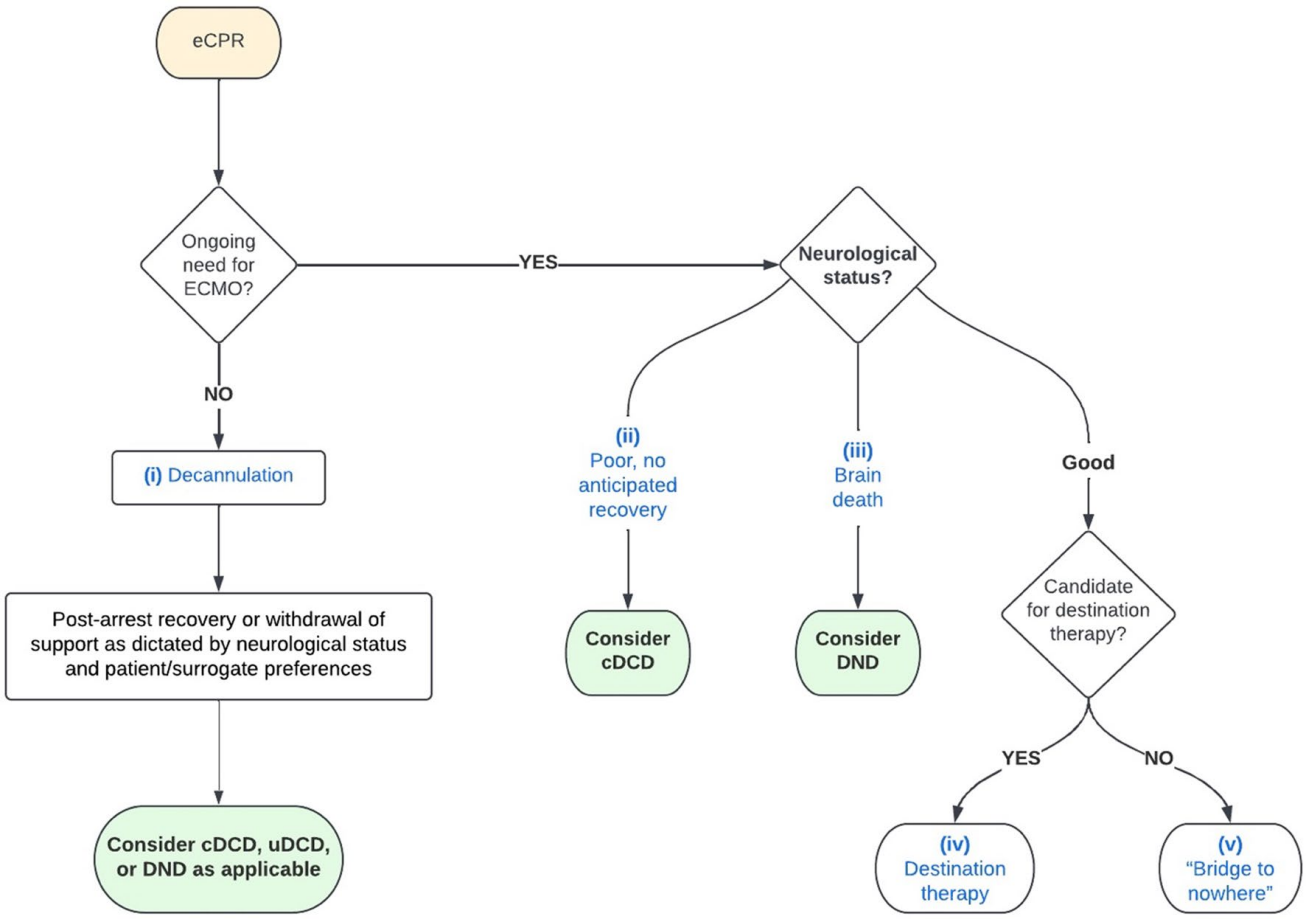
Outcome categories after successful cannulation for eCPR. *eCPR* extracorporeal cardiopulmonary resuscitation; *ECMO* extracorporeal membrane oxygenation; *cDCD* controlled donation after circulatory death; *uDCD* uncontrolled donation after circulatory death; *DND* donation after neurological death. Broad outcome categories after initiating eCPR are depicted in blue font; transplant recovery considerations are depicted in green filled ovals. One possible outcome is (i) decannulation following return of spontaneous circulation, leading to post-arrest care akin to standard CPR. For those unable to be weaned from ECMO, outcomes include: (ii) poor neurological function without anticipated recovery; (iii) death by neurologic criteria (“brain death”); (iv) good neurological function with eligibility for destination therapy (e.g., left ventricular assistive device or transplantation); and (v) good neurological function with inability to wean from ECMO and ineligibility for destination therapy (i.e., “bridge to nowhere”)

Offering eCPR to eligible patients could increase survival, but long-term outcomes are uncertain. For patients who can be weaned from ECMO support, potential outcomes include survival with good neurological outcomes with or without ongoing standard cardiopulmonary support (e.g., mechanical ventilation, medications) and survival with devastating neurological outcomes and/or other significant morbidities (Fig. 3). eCPR guidelines lack consensus on duration of therapy after which futility can be determined. Current standards suggest three to four days are sufficient, though there are reported durations up to eight days [15, 46]. Consensus should be developed based on parameters that can drive futility determinations, including validated markers of multiorgan failure or monitoring to determine extent of neurological injury [47]. Consistency and transparency are critical for public trust and fair utilization of scarce resources. It is also vital to prevent public misconceptions that the availability of ECMO for NRP might influence decisions to terminate ECMO for cardiopulmonary support.

### Risk of complications

If ECMO programs disseminate, circulatory determination of death for select patients may depend upon eCPR availability, efficacy, and effectiveness. But sufficient data are not available to conclusively determine which patients will benefit from eCPR compared to conventional resuscitation. Consequently, risks of major ECMO complications carry particular weight in patients who might have survived with good neurological outcomes with conventional CPR. These patients might needlessly be exposed to infection, cardiac or intracranial injury, and significant vascular events, such as bleeding, hemolysis, and thrombosis [25]. Although early data from high-performing eCPR programs suggest improved survival with CPC score of 1 or 2 in appropriately selected patients [8, 18], the potential for serious adverse events requires frank discussions among the clinical team and, importantly, with patients and their surrogate decision-makers.

These risks also underscore the need for evidence-based determinations regarding which patients are likely to benefit from eCPR. As eCPR is increasingly studied and employed, growing data may elucidate patient characteristics that point to better or decreased likelihood of benefit. In turn, eCPR should not and will not be offered to all patients, intentionally excluding those who are deemed more likely to be harmed or suffer significant adverse events than to benefit from this mode of resuscitation.

### Bridge to nowhere

It is plausible that a unique subset of patients dependent upon ECMO for cardiopulmonary support will have the capacity and ability to participate in their own care decisions, yet be clinically ineligible for destination therapies (e.g., ventricular assist devices, heart transplantation; Fig. 3). The initiation of ECMO in eCPR is intended as a bridging therapy and this ethically and emotionally fraught scenario has therefore been termed the “bridge to nowhere” [48]. The incidence of these cases is unknown since they are likely approached on a case-by-case basis by individual intensive care units and ethics consultation committees. Though likely to occur rarely with eCPR, protocols must include contingency planning and discussions regarding the potential for this scenario with patients and/or their decision-makers.

Clinical teams, along with palliative care consultation and input from ethics committees, usually have the experience to navigate the difficult scenarios in which there is disagreement between clinicians and surrogate decision-makers about withdrawal of life-sustaining treatment from a patient lacking decisional capacity. These conflicts usually prompt multidisciplinary meetings with the patient’s next-of-kin in which values and priorities are discussed to allow for resolution through shared decision-making. However, a similar disagreement between the clinical team and the patient themself regarding withdrawal of life-sustaining treatment is relatively novel, more complicated, and potentially more morally distressing for those involved. Therefore, centers that develop eCPR programs should also be required to develop mechanisms for navigating the likely rare, but possible, “bridge-to-nowhere” scenario. Healthcare institutions must prepare to address these complex cases in real time with early involvement of a multidisciplinary team that includes palliative care, psychiatry, and ethics consultants [49].

## Considerations for cDCD after eCPR

### Permanence versus irreversibility

The Uniform Determination of Death Act (UDDA), which informs laws enacted by all states, delineates the legal definition of death. Death determination in the USA requires irreversible cessation of either cardiopulmonary functions or all functions of the entire brain [50]. Irreversible cessation means that these functions *cannot* be resumed, regardless of intervention. In practice, however, death is declared after *permanen*t cessation of cardiopulmonary or neurologic function; permanence means that these functions will not resume spontaneously and no measures to reverse cessation will be performed because doing so would be clinically futile or unaligned with the patient's goals of care [51]. The UDDA was originally written in 1980, and the drafters of the legal definition of death likely did not foresee the use of ECMO technology for organ preservation or the ability to perform regional perfusion by preventing circulation to the brain. This has prompted active discourse about whether precluding blood flow to the brain while reinstating circulation to select parts of the body makes clinicians complicit in a patient’s death or undermines the death determination [36, 52].

But crucially, the patient is already dead in all DCD protocols, including those that use NRP. Prior to any DCD, the patient is declared dead after circulatory cessation according to clinical and ethical standards. The significance of circulatory death is the permanent discontinuation of oxygenated circulation to the brain, leading to eventual irreversible and complete loss of all brain function [51]. Effective postmortem occlusion of brain circulation prior to initiating NRP, monitored with cerebral oximetry, ensures that only organs potentially eligible for recovery are perfused without impeding the natural progression to complete loss of brain function [53]. In NRP, successful occlusion of blood flow to the brain ensures that circulation only results in organ perfusion, not resuscitation; the deceased patient remains dead [39, 54]. In effect, this is similar to ex vivo perfusion, in which perfusion is restored to the recovered organ to increase transplant viability, while the process towards loss of brain function in the donor body is allowed to continue.

### Autoresuscitation

All DCD protocols require a “hands-off” period after circulatory and respiratory cessation following termination of lifesaving efforts. This designated period of time is meant to ensure autoresuscitation of the heart does not take place prior to organ preservation and recovery. Studies examining prevalence of autoresuscitation are hindered by biased estimation, but available evidence suggests it is incredibly unlikely after five minutes in adults [55]. Nonetheless, protocols for DCD vary considerably in designated hands-off times, ranging from two to ten minutes [56]. This variability demonstrates disagreement on when potential donors meet the definition of circulatory cessation necessary to satisfy legal requirements of death. For dissemination of the DCD protocols described here, there should be national consensus on the autoresuscitation period, leading to uniform practice [53].

## Considerations for NRP programs for uDCD

### Ethical permissibility of performing NRP without eCPR

Having NRP programs operate within systems lacking eCPR capability would exacerbate concerns about unethical prioritization of organ donation over lifesaving efforts [57, 58]. Imagine a patient in cardiac arrest just outside of an eCPR catchment area is brought to an organ preservation facility that utilizes NRP, despite having been eligible for eCPR if available. This patient would become an organ donor when their life might have been saved with the same technology. The injustice of this resource allocation may be deepened by eCPR programs being disproportionately available in wealthier, less diverse communities. To alleviate the potential for such inequities and public mistrust, NRP programs in the USA should only exist within healthcare systems with eCPR capability.

When resources or personnel are limited, use of ECMO for preserving organs should never interfere with care for patients who could benefit from ECMO use for lifesaving purposes [38]. However, a patient deemed clinically ineligible for eCPR should not automatically be excluded from NRP after determination of death [38]. In other words, after it is determined that a patient with cardiac arrest refractory to standard resuscitation would not benefit from eCPR, the priority should shift to maximal respect for that individual’s potential wishes to be an organ donor by optimizing preservation of transplantable organs after death determination. As above, public perceptions must be managed to understand that eCPR allocation decisions are rooted in evidence-based determinations of clinical indication and are never affected by organ donor designation. Wherever possible, clinical teams providing life-saving care and participating in determination of death should be separate from personnel involved in donation discussions and organ recovery to avoid real or perceived conflicts of interest [59].

### Organ donation decisions

Similar to the inclusion of eCPR in end-of-life care discussions, potential use of NRP should be disclosed with sufficient transparency so persons who consider authorizing donation can make informed decisions. In addition to selecting whether to authorize tissue and/or solid organ donation, donor registries and advance directives could include additional selections for willingness to be placed on NRP if indicated. Monitoring trends in do-not-resuscitate designations and organ donor authorization rates concurrent with eCPR and NRP dissemination are critical to gauge community acceptance and modify protocols accordingly.

### Permission to preserve

Integrated eCPR/NRP protocols have potential to maximize lives saved, but successful examples in countries such as Portugal and Italy are not directly translatable to the USA, in part because of the substantial difference in organ donation frameworks [5–7]. In countries such as Portugal, where organ donation decisions are managed through presumed consent, or an opt-out framework, individuals are treated as willing organ donors unless they have registered wishes to the contrary [6]. While family members are still frequently consulted in ultimate decisions of organ donation, the presumed consent framework allows interventions for the purposes of organ preservation and recovery—for example, cannulation and initiation of NRP—to proceed promptly.

In the opt-in framework in the USA, individuals are not presumed to be willing organ donors unless they have indicated this desire through first-person authorization (e.g., enrollment in state registries) or organ donation is considered to be in accordance with their wishes by their surrogate decision-makers. The opt-in framework therefore requires additional assurances in pursuing NRP for organ preservation and recovery. For cases with first-person authorization for organ donation, cannulation and NRP could be initiated soon after the “hands-off” period. In unexpected cardiac arrests without evidence of first-person authorization, decision-makers should be asked for permission to preserve organs of the deceased via NRP prior to its initiation. This step should be separated from a subsequent request for donation authorization [60]. The loved ones of the deceased may be distressed or traumatized and might need more time to decide whether the deceased would have wanted to donate organs. Preserving the potential donor’s organs via NRP with either first person or decision-maker authorization also allows time for an organ procurement organization representative to arrive, maintaining a helpful distinction between patient care and organ donation teams. If, after discussion, the decision-maker decides not to authorize donation, preservation would promptly be discontinued.

## Recommendations

To realize benefits from disseminating eCPR programs and implementing integrated eCPR/NRP protocols in the USA, ethical considerations must be addressed proactively. Relevant legislation, oversight mechanisms, and public engagement often lag behind clinical implementation of emerging technologies, potentially creating ethical, legal, and social gaps that need to be addressed retroactively. Therefore, our position is that anticipating these gaps and beginning the work to address them must occur concurrently with clinical investigation of these novel protocols. Considering the most pressing issues, we propose the following recommendations for equitable and just protocol implementation:

1. National data from use of ECMO for resuscitation and organ preservation should be centralized and methodically monitored, similar to quality measures for interventional procedures for other time-sensitive emergencies such as large-vessel-occlusion stroke and ST-elevation myocardial infarction. While consortia databases exist, such as those data maintained by the Extracorporeal Life Support Organization (ELSO) [61], more comprehensive data are required along with uniform definitions and standardized collection. Centers implementing eCPR and/or NRP programs should agree to participate in the centralized data repository. An imperative to publish or otherwise disseminate protocols for inter-institutional collaboration is likewise essential to drive evidence-based practice.
2. Data should be evaluated iteratively to develop consensus guidelines on a number of ECMO-related factors, including eCPR eligibility parameters based on likelihood of benefit, clinical markers of futility that warrant discontinuation of ECMO, and agreement on duration of the necessary “hands off” period. Major clinical societies with relevant expertise among their membership, such as the American Thoracic Society, American College of Chest Physicians, American College of Emergency Physicians, and Society of Critical Care should form consortia or working groups to develop consensus for clinical parameters to guide uniform practice in eCPR initiation and termination.
3. Healthcare institutions should not establish NRP uDCD programs until eCPR programs are in place. Once robust data are available to select candidates appropriate for eCPR, it is ethically permissible for an institution capable of eCPR and NRP to proceed with uDCD without offering eCPR based on clinical eligibility. Institutions should also have committees in place for monitoring care patterns and outcomes, with the agility to respond to difficult cases in real time.
4. The legal definition of death should be reexamined to account for advances in medical practice and technology [54], including the use of ECMO for NRP.
5. While informed consent discussions may be justifiably limited in emergent situations [62], there should be concerted efforts to include patients and/or surrogate decision-makers in every interventional decision as soon as logistically possible. Informed consent for eCPR or permission for NRP should be treated as a process, rather than static decision, with an emphasis on transparency and effective, regular communication.
6. Coordinated efforts must be undertaken to assess and address relevant public perceptions with appropriate community engagement. Such efforts should include community education to promote understanding of the role of ECMO in resuscitation and organ preservation. Efforts should also emphasize that protocols will always prioritize lifesaving over organ donation considerations, addressing the potential misperception that organ donor designation could impact care decisions. Community outreach must likewise address evolving issues around end-of-life wishes, including novel considerations pertinent to use of eCPR and NRP. Engaging community stakeholders early as partners in protocol design, implementation, and evaluation can help ensure eCPR/NRP program acceptance and sustainability [63].

## Conclusion

It is in the public’s interest for the USA to investigate and develop best practices for the dissemination of eCPR programs and the integration of eCPR protocols with NRP organ preservation. This can be accomplished ethically if implementation occurs with great care to optimize clinical outcomes based on evolving evidence, to distribute resources justly, and to promote informed healthcare decisions that align with patients’ values. If successful, integrated eCPR and NRP protocols have great potential to maximize lives saved through both improved resuscitation with good neurological outcomes and substantially increased organ donation opportunities when resuscitation is unsuccessful or not in accordance with individuals’ wishes.

## Data Availability

Not applicable.

## Abbreviations

cDCD: Controlled donation after circulatory death
CPC: Cerebral performance category
CPR: Cardiopulmonary resuscitation
DCD: Donation after circulatory death
DND: Donation after neurological death
ECMO: Extracorporeal membrane oxygenation
eCPR: Extracorporeal cardiopulmonary resuscitation
NRP: Normothermic regional perfusion
uDCD: Uncontrolled donation after circulatory death
UDDA: Uniform Declaration of Death Act
